# Associations between Retinal Markers of Microvascular Disease and Cognitive Impairment in Newly Diagnosed Type 2 Diabetes Mellitus: A Case Control Study

**DOI:** 10.1371/journal.pone.0147160

**Published:** 2016-01-15

**Authors:** Vasanth Venkat Naidu, Khalida Ismail, Stephanie Amiel, Reena Kohli, Roxanne Crosby-Nwaobi, Sobha Sivaprasad, Robert Stewart

**Affiliations:** 1 King’s College London, London, United Kingdom; 2 Kent and Medway NHS and Social Care Partnership Trust, Maidstone, United Kingdom; 3 NIHR Moorfields Biomedical Research Centre, London, United Kingdom; University of Florida, UNITED STATES

## Abstract

**Objective:**

To investigate associations between retinal microvascular changes and cognitive impairment in newly diagnosed type 2 diabetes mellitus.

**Design:**

Case control study.

**Setting:**

A primary care cohort with newly diagnosed type 2 diabetes mellitus.

**Methods:**

For this analysis, we compared 69 cases with lowest decile scores (for the cohort) on the Modified Telephone Interview for Cognitive Status and 68 controls randomly selected from the remainder of the cohort. Retinal images were rated and the following measures compared between cases and controls: retinal vessel calibre, arterio-venous ratio, retinal fractal dimension, and simple and curvature retinal vessel tortuosity.

**Results:**

Total and venular (but not arteriolar) simple retinal vessel tortuosity levels were significantly higher in cases than controls (t = 2.45, p = 0.015; t = 2.53, p = 0.013 respectively). The associations persisted after adjustment for demographic factors, retinopathy, neuropathy, obesity and blood pressure. There were no other significant differences between cases and controls in retinal measures.

**Conclusions:**

A novel association was found between higher venular tortuosity and cognitive impairment in newly diagnosed type 2 diabetes mellitus. This might be accounted for by factors such as hypoxia, thrombus formation, increased vasoendothelial growth factor release and inflammation affecting both the visible retinal and the unobserved cerebral microvasculature.

## Introduction

Type 2 diabetes mellitus (T2DM) is associated with a range of adverse consequences, including a higher risk of cognitive impairment and dementia, which is increasingly becoming recognised [1–3]. It has been estimated that the risk of developing Alzheimer’s disease is doubled and that of vascular dementia tripled in T2DM [4]. The pathways underlying these associations may be multiple. Pre-diabetes factors such as obesity, or other co-morbidities such as hypertension and dyslipidemia, may play a role, and diabetic effects on large and small vessels serving the brain are also likely [5].

Retinal microvascular abnormalities in T2DM range from mild, non-proliferative changes to proliferative diabetic retinopathy, and reflect the severity of the condition and the degree of glycemic control. [6]. Retinal vasculature shares embryological origins, physiological characteristics, structure and size with cerebral vasculature [7, 8], and thus provides a potential means to investigate further the relationship between diabetes and cognitive function. Investigations into associations between retinal imaging measurements and cognitive function in T2DM have been relatively few. Kadoi and colleagues [9] found that diabetic retinopathy was associated with cognitive impairment 6 months after coronary artery bypass surgery, and Ding and colleagues [10] found that diabetic retinopathy was independently associated with lifetime cognitive decline in older men. However, a recent study by Crosby-Nwaobi and colleagues [11] found that people with proliferative diabetic retinopathy had less cognitive impairment than those with mild or no retinopathy, suggesting that the increased incidence of cognitive impairment in T2DM may be due to other factors rather than microvascular disease reflected in the retina.

A limitation with previous research in this field has been that T2DM cases often include people with long diabetes duration which might give rise to obscured relationships due to selective survival, as well as confounding by other factors co-occurring during disease progression. In a sample of newly-diagnosed T2DM we sought to compare measurements derived from retinal imaging between people with and without cognitive impairment close to the disease onset.

## Materials and Methods

### Cases and Controls

The analysed samples were drawn from baseline participants in the South London Diabetes Study (SOUL-D), an ongoing prospective study of people with newly diagnosed T2DM [12]. Ethical approval was granted by the King’s College Hospital Research Ethics Committee (reference 08/H0808/1) and by Lambeth, Southwark and Lewisham Primary Care Trusts (reference RDLSLB 410) and all participants gave written informed consent, including access to their medical records. The SOUL-D study was carried out to identify individuals with newly diagnosed T2DM in order to investigate associations of a range of biopsychosocial factors with biomedical outcomes over a 2-year period. The setting comprised adjacent London boroughs of Lambeth, Southwark and Lewisham–a multi-ethnic and socioeconomically diverse catchment population of approximately 0.75 million residents–and the sampling frame included all 138 general practices in this area, 96 of which agreed to participate. Participants were aged 18–75 years and recruited from these primary care centres. All participants had been diagnosed with T2DM within the last six months. Exclusion criteria comprised: i) diabetes other than T2DM; ii) temporary residence and/or residence outside the catchment area; iii) lack of fluency in English; iv) movement from another primary care team; v) a terminal or separate advanced condition; vi) severe mental illness (dementia, substance dependence, bipolar disorder, personality disorder); and vii) severe advanced complications of diabetes (blindness, requiring dialysis or having undergone an above-the-knee amputation). Of 1200 participants recruited at the time of the analyses carried out for this paper, 1084 (90.3%) had received cognitive assessment using the modified Telephone Interview for Cognitive Status (TICSM), a widely used measure of global cognitive function [13, 14]. For this study, cases were defined as SOUL-D participants with TICSM scores in the lowest 10% of the sample distribution (score 17 or below), and a randomly selected sample of remaining participants were identified as controls. Case/control status was blinded on the database until retinal measurements had been carried out. Retinal imaging had been performed on all SOUL-D participants at baseline, but vascular parameters had not been measured. Because of limited resource for vascular measurements, a case control approach was felt to be most time-efficient, comparing participants with and without cognitive impairment. Retinal images were therefore retrieved for further grading, with continued sampling from the SOUL-D database carried out until images of sufficient quality were available for 69 cases and 68 controls.

### Measurements

**Fig 1:**
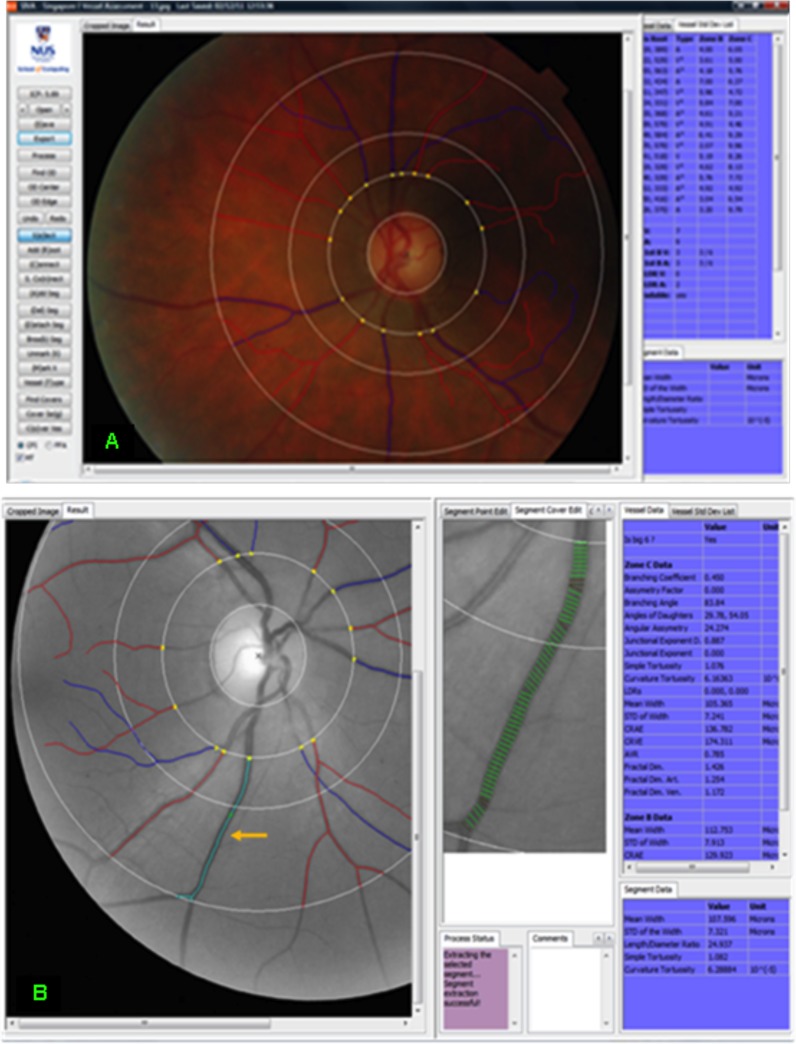
Colour and grey-scale fundus photographs of two different eyes analysed by SIVA. (A) Colour fundus photograph showing optic disc, three concentric subzones, and arterioles and venules, with vessel data. (B) Grey-scale fundus photograph with screen showing multiple measurements of the diameter of the highlighted vessel and vessel data.

Retinal photography had been performed following a standardized protocol [15]. In brief, stereoscopic retinal photographs, after pupil dilation, were taken of both eyes from patients on the SOUL-D database, using a Topcon Fundus Camera (TRC 50-VT; Tokyo Optical, Tokyo, Japan). Retinal images were graded by Singapore I Vessel Assessment (SIVA), a semi-automated program developed at the National University of Singapore. Excluded images were those with incomplete representation of Zone C or excessive blurring, or if there were fewer than four arterioles or venules that could be graded. The retinal images selected were those of best quality from either the right or left eye and were the closest taken to within 6 months of the date of recruitment to SOUL-D. SIVA automatically detected the optical disc if the image was of sufficient quality; otherwise a trained grader manually detected it. After optical disc identification, SIVA constructed a grid with three concentric subzones, 0.5 (zone A), 1.0 (zone B) and 2.0 (zone C) optic disc diameters from the optic disc margin at the centre. The grader then commanded the program to find all vessels, and arterioles and venules were detected. SIVA traced the vessel using its centreline, from zone B to zone C (Fig 1). SIVA has an ability of 70–90% of detecting appropriate arterioles and vessels [16], but if the grader deemed the detection of vessels false or inaccurate, they were manually corrected. This was based on parent vessels, and cross-over and colour of vessels. The grading of each image took approximately 1–1.5 hours. The grader was trained and tested by a senior grader. Inter-and intra-grader reliability for retinal measurements, tested with 10 random images, gave interclass correlation coefficients ranging from 0.88–0.99.

The measurements made were retinal vessel caliber, arterio-venous ratio, retinal fractal dimension and retinal vessel tortuosity. Retinal vessel calibers were defined via the central arteriole equivalent and central venule equivalent as described by the Parr-Hubbard-Knudtson formula [17] and the arterio-venous ratio was calculated from the ratio between these respective measures. These measurements were based on the widest six arterioles and venules. The retinal fractal dimension, quantifying the degree of complexity of the branching of the retinal vasculature [18], was calculated for all vessels, and then separately for arterioles and venules. Retinal tortuosity [19] was measured by SIVA in two respects: i) simple tortuosity, based on estimating the difference between the actual length of the vessel segment, incorporating the curves, and the straight length of the vessel segment, divided by the straight length of the vessel segment (i.e. arc length/chord length); ii) curvature tortuosity, based on vessel curvature and, unlike simple tortuosity, taking into account increased length due to bowing and multiple points of inflexion (calculated from the integral of the curvature square along the vessel path, normalised by the total length of the path) [20]. Simple and curvature tortuosity were quantified for all vessels with a width larger than 40 μm coursing through from Zone B to Zone C, and for arterioles and venules separately.

Covariates obtained from baseline SOUL-D examinations and used in this analysis comprised the following: age, sex, ethnicity (white, black, Asian/other), body mass index, resting systolic and diastolic blood pressure, receipt of antihypertensive medication, retinopathy (defined as any retinal haemorrhage or microaneurysm found in either eye by an ophthalmologist), neuropathy (defined on the basis of a vibration perception threshold score of 25 or more) and HbA1c.

### Statistical Analysis

Cases and controls were first described in relation to covariates. Mean and standard deviation (SD) scores for retinal vessel measures were then compared between the two groups, using t-tests to assess levels of statistical significance. Significant and near-significant (p<0.10) differences were then assessed further for independence in sequentially adjusted linear regression models: adjusting first for demographic factors (age, sex, ethnicity); next for retinopathy, neuropathy and BMI; next for blood pressure and antihypertensive medication; and finally for HbA1c. All analyses were carried out using Statistical Package of the Social Sciences (SPSS) software, version 20.

## Results

Sample characteristics are summarised in Table 1. Cases were significantly more likely to be female but there were no other significant differences.

**Table 1:** Sample characteristics.

Characteristic		Percentage/mean (SD)		Chi-square (df) or t-statistic, p-value
		Controls (n = 68)	Cases (n = 69)	
**Age group (%)**	<50	32.4	26.1	2.2 (1), 0.14
	50–59	44.1	29.0	
	60–69	14.7	23.2	
	70+	8.8	20.3	
**Female sex (%)**		35.3	55.1	5.4 (1), 0.020
**Ethnic group (%)**	White	51.5	42.0	1.7 (2), 0.42
	Black	36.8	47.8	
	Asian/Other	11.8	10.1	
**BMI group (%)**	<30.0	54.4	50.7	0.2 (1), 0.66
	30.0–34.9	20.6	24.6	
	35.0–39.9	17.6	13.0	
	40.0+	7.5	11.6	
**Antihypertensive medication (%)**		43.3	49.3	0.5 (1), 0.49
**Neuropathy present (%)**		11.8	13.0	0.5 (1), 0.82
**Retinopathy present (%)**		11.9	22.4	2.6 (1), 0.11
**Mean (SD) systolic blood pressure (mmHg)**		137 (13.7)	136 (19.7)	0.27, 0.788
**Mean (SD) diastolic blood pressure (mmHg)**		83 (9.5)	81 (9.9)	1.25, 0.213
**Mean (SD) HbA1c (%)**		6.9 (1.1)	7.2 (1.6)	1.1, 0.264

Retinal measures are compared between the case and control groups in Table 2. There were significantly higher simple total and venular tortuosity measures in cases compared to controls but no differences in other measures.

**Table 2:** Retinal vascular marker distributions in cases and controls.

Retinal vascular marker	Mean (SD) in Controls (n = 68)	Mean (SD) in Cases (n = 69)	Mean difference (95% CI)	t-statistic (p-value)
**Arteriolar calibre**	146.9 (18.5)	150.3 (15.6)	3.3 (-2.4, 9.1)	1.14 (0.26)
**Venular calibre**	216.9 (26.5)	218.6 (22.2)	1.7 (-6.6, 9.9)	0.40 (0.69)
**Arteriovenous ratio**	0.68 (0.06)	0.69 (0.05)	0.01 (-0.01, 0.03)	1.07 (0.29)
**Fractal Dimension:**				
**Arterioles**	1.22 (0.06)	1.23 (0.06)	0.01 (-0.01, 0.03)	0.95 (0.35)
**Venules**	1.20 (0.05)	1.21 (0.05)	0.01 (-0.01, 0.03)	1.37 (0.17)
**Total**	1.43 (0.05)	1.44 (0.05)	0.01 (-0.01, 0.03)	1.38 (0.17)
**Simple Tortuosity:**				
**Arterioles**	1.100 (0.02)	1.107 (0.03)	0.006 (-0.003, 0.015)	1.27 (0.21)
**Venules**	1.102 (0.02)	1.114 (0.03)	0.012 (0.002, 0.021)	2.53 (0.013)
**Total**	1.102 (0.02)	1.110 (0.02)	0.009 (0.002, 0.016)	2.45 (0.015)
**Curvature Tortuosity (x10**^**6**^**)**				
**Arterioles**	74.0 (32.6)	71.6 (31.2)	-2.5 (-13.2, 8.4)	-0.44 (0.66)
**Venules**	78.5 (30.5)	80.4 (21.1)	1.9 (-6.9, 10.8)	0.43 (0.67)
**Total**	76.4 (27.5)	76.1 (24.6)	-0.3 (-9.1, 8.5)	-0.06 (0.95)

In multivariable models (Tables 3 & 4), the associations with greater simple venular and total tortuosity persisted and were largely unaltered following adjustment for covariates. To investigate further age as a potential confounder, we analysed associations with these outcomes in the combined samples of cases and controls, modelling the simple tortuosity measures as dependent variables in linear regression analyses with age as the independent variable; in summary, associations with older age were, if anything, negative rather than positive and were not substantial in strength (for 10-year age increments with simple arteriolar tortuosity: B -0.004, 95% CI -0.008 to 0, p = 0.052; with simple venular tortuosity: B -0.001, 95% CI -0.005 to 0.003, p = 0.489; with simple total tortuosity: B -0.003, 95% CI -0.006 to 0, p = 0.087).

**Table 3:** Linear regression analysis of the association between cognitive impairment and simple venular tortuosity.

Sequential adjustments	B-coefficient (95% CI)
Unadjusted	0.012 (0.003, 0.021)
Adjusted for age, sex, ethnic group	0.012 (0.002, 0.021)
Further adjusted for retinopathy, neuropathy, BMI	0.013 (0.003, 0.023)
Further adjusted for systolic blood pressure, diastolic blood pressure and antihypertensive medication	0.014 (0.003, 0.024)
Further adjusted for HbA1c	0.013 (0.002, 0.025)

**Table 4:** Linear regression analysis of the association between cognitive impairment and simple total tortuosity.

Sequential adjustments	B-coefficient (95% CI)
Unadjusted	0.009 (0.002, 0.016)
Adjusted for age, sex, ethnic group	0.009 (0.002, 0.017)
Further adjusted for retinopathy, neuropathy, BMI	0.009 (0.001, 0.016)
Further adjusted for systolic blood pressure, diastolic blood pressure and antihypertensive medication	0.010 (0.002, 0.018)
Further adjusted for HbA1c	0.011 (0.003, 0.020)

## Discussion

In this case control study carried out within a sample of people with newly diagnosed type 2 diabetes, participants with cognitive impairment (defined as the 10% lowest scores on a commonly used test of global function) and randomly selected controls from the same cohort were identified. A series of measurements derived from retinal imaging was then compared between the two groups, ascertained blind to case/control status. While most retinal image measures did not differ between the comparison groups, simple vessel tortuosity was higher in cases than controls, an association which reflected higher simple venular, rather than arteriolar, tortuosity. To our knowledge, this is a novel finding.

The strengths of the study are that cases and controls were drawn from the same source sample, limiting the likelihood of selection bias, and the blind ascertainment of retinal measures, reducing information bias. A range of potential confounding factors were considered, but did not appear to account for the associations found (and age, specifically, was not associated with either of the outcomes found to be associated with cognitive impairment). In considering the findings, it is important to bear in mind that the source SOUL-D study recruited people newly diagnosed with T2DM. This was methodologically advantageous in minimising the likelihood of confounding and/or reverse causality relating to longer-lasting or more advanced diabetes. However, generalisability cannot be assumed to people with diabetes of longer duration; for example, it is possible that the negative findings for retinopathy and some of the retinal measures might have been a result of the restriction to newly diagnosed cases. Considering other limitations, the case and control sample sizes were relatively small, and absent associations should be treated with caution as there might have been smaller but clinically relevant differences which the study did not have statistical power to identify. It should also be borne in mind that simple venular and total tortuosity were only two of twelve measures analysed which differed significantly between cases and controls, and that the findings although plausible require replication.

In our study, although twice as many cases compared to controls had clinically recognised retinopathy, the numbers in this cohort were small in both groups, and the association between simple venular tortuosity and cognitive impairment remained significant after adjustment, suggesting that vessel tortuosity was not simply a marker of retinopathy. While one recent study found that venular and arteriolar curvature tortuosity was decreased in proliferative diabetic retinopathy [21], no association was found in another between venular tortuosity and diabetic retinopathy [22]. Our observation may therefore indicate a different underlying mechanism. Hypertension has also been found to be associated with increased venular tortuosity [23], shares risk pathways with diabetes [24], and is an independent risk factor for cognitive decline and dementia [25]. However, there was no significant difference in blood pressure levels or treated hypertension between cases and controls in our study, and adjustment for these factors had little impact on the association between simple venular tortuosity and cognitive impairment.

Our finding that curvature venous tortuosity showed no association with increasing levels of cognitive impairment is potentially anomalous as it is considered a more robust measure of vessel tortuosity than simple tortuosity, taking into account increased length due to bowing and multiple points of inflection [20]. Despite this, there are still potential reasons why increased simple tortuosity alone might link with cognitive impairment. One of these is that venular tortuosity might reflect underlying hypoxia. Retinal venular tortuosity has been found to be increased in high altitude conditions [26, 27], potentially because of increased shear stress causing elongation of vessel segments [27] as a result of increased blood flow to correct local hypoxia. In diabetes, hyperglycemia may cause hypoxia by various mechanisms such as vasoconstriction, leucostasis and induction of a pro-inflammatory state [28], as well as by disrupting blood flow via vascular endothelial and basement membrane disruption [29]. Therefore brain hypoxia causing cognitive impairment in diabetes might also result in venular tortuosity visible in retinal imaging. Venular tortuosity has also been found to be associated with increased venous thrombus formation and small venule strokes [30], which may in turn lead to decreased blood flow and brain and cognitive dysfunction [31]. Both hypoxia and hyperglycemia are associated with increased vasoendothelial growth factor release, which stimulates vasculogenesis and angiogenesis in response to hypoxia [32]; these processes might initiate retinal changes in early diabetes, localized to the venules and capillaries of the superficial inner retinal vasculature [33], resulting in venular tortuosity [34], and may increase vascular permeability [35], leading to protein extravasation, chronic edema and tissue necrosis. Finally, increased high-sensitivity C-reactive protein has been found to be associated with increased retinal venular tortuosity [36] and the finding might thus reflect an underlying pro-inflammatory state implicated in T2DM [37], cognitive decline [38] and dementia [39], separate from hyperglycaemia.

Our findings suggest at least a link, whether causal or not, between retinal microvascular change and cognitive impairment in T2DM. A recent review concluded that retinal microvascular changes showed consistent and moderately strong associations in cross-sectional studies with dementia, with relative cognitive impairment in people without dementia, and with brain imaging abnormalities [40]. On the other hand, the few longitudinal studies only showed marginal associations with dementia or cognitive decline, but more consistent links with progression of brain imaging abnormalities: relatively weak for vascular caliber measurements, intermediate for arteriovenous nicking and focal narrowing, and strong for retinopathy. These associations were also stronger in people with both hypertension and diabetes. This suggests that increased damage to retinal vessels is associated with increased cognitive impairment. However, not all studies have supported this [11, 41, 42]. In a sample of middle aged people without stroke, retinopathy was associated with poorer cognitive function, but smaller AVR was not [43].

Our study supports the contention that retinal vasculature images are worth considering as potential risk markers for cognitive decline in people with diabetes, potentially at a relatively early stage after diagnosis. The link with cognitive function might well be independent of retinopathy, possibly reflecting underlying mechanisms implicated in both T2DM and cognitive impairment, or underlying mechanisms in T2DM increasing the risk of cognitive impairment. Further research is needed to confirm our findings, to ascertain the extent to which these measures predict cognitive decline prospectively, and to ascertain factors accounting for the observed associations.

### Strengths and Limitations

- To our knowledge, this study is the first to investigate associations between measurements derived from retinal images and cognitive function in newly diagnosed T2DM.
- Cases and controls were drawn from a single cohort and retinal images were rated blind to case status.
- The sample size was limited and negative findings should be viewed with caution.
- Manual correction of vessel detection potentially subjected the study to a larger degree of measurement error
- The study was exploratory in nature and the association with venular tortuosity requires replication in an independent sample.

## Supporting Information

S1 Dataset Patient data from the variables used in the study. (XLSX)
